# Papillary Thyroid Carcinoma Presenting as Multinodular Goiter With Possible Metastasis: A Case Study

**DOI:** 10.7759/cureus.97589

**Published:** 2025-11-23

**Authors:** Aron Yunatanov, Tzipora Levitt, Tiffany K Tran, Andrew Zalot, Jonathan Gulkarov, Rafael Ilyaev, Avraham Izrailov, Stella Ilyayeva

**Affiliations:** 1 Medicine, New York Institute of Technology (NYIT) College of Osteopathic Medicine, Old Westbury, USA; 2 Medicine, Hofstra University, Hempstead, USA; 3 Medicine, St. John's University, Queens, USA; 4 Medicine, New York Institute of Technology (NYIT), Old Westbury, USA; 5 Endocrinology and Diabetes, Atlantic Endocrinology and Diabetes, Queens, USA

**Keywords:** extrathyroidal extension, fine-needle aspiration, lung metastasis, multinodular goiter, papillary cancer of thyroid

## Abstract

Multinodular goiter (MNG), which may be caused by a variety of risk factors, is typically incidentally found. It is not uncommon for an incidental finding of MNG to lead to a diagnosis of papillary thyroid carcinoma (PTC); however, close monitoring for metastasis is necessary in high-risk patients. Herein, we present the case of a 71-year-old male, seemingly following a normal path of incidental discovery of MNG leading to PTC upon thyroid ultrasound and fine-needle aspiration (FNA) biopsy diagnostic exams. A total thyroidectomy was performed, with pathology confirming an aggressive tumor type (tall cell variant, T4a). Postoperative findings revealed extrathyroidal extension (ETE) with possible lung metastasis. Complications such as these highlight the importance of closely monitoring patients with a higher risk of aggressive thyroid cancer.

## Introduction

Multinodular goiter (MNG) is a disease of the thyroid glands, typically characterized by the presence of thyroid nodules, and can be classified as either toxic or nontoxic [1]. Nontoxic MNG does not usually cause biochemical abnormalities of the thyroid gland; however, toxic MNG can produce thyroid hormone, which may lead to hyperthyroidism [2]. MNGs appear to be asymptomatic most of the time, usually being discovered incidentally upon a routine physical exam or assessments conducted for unrelated conditions [2]. Typically, a nontoxic MNG is found in a euthyroid patient, referring to normal function of the thyroid gland with no abnormal findings in the patient’s history or in clinical examination, usually confirmed after hormonal exams are done [1]. In extreme cases, the MNG may grow to cause compressive symptoms, which can include difficulty breathing, swallowing, and/or a choking sensation [2]. Once found or suspected, a thorough history is important to ascertain the rate of thyroid growth, risk factors for thyroid cancer, family history of goiter, hoarseness, and symptoms of hyperthyroidism [2]. Laboratory tests (to measure thyroid-stimulating hormone (TSH)), imaging studies such as thyroid ultrasound, and/or fine-needle aspiration (FNA) biopsy are performed to better understand the features of the thyroid nodule [1]. Both FNA and ultrasound have limited accuracy in identifying malignancy; however, this risk is accepted when both are used in conjunction to aid each other [1]. 

A blood test measuring TSH levels allows a physician to quickly differentiate between toxic and nontoxic MNG [1]. In nontoxic MNG patients, TSH levels are typically normal or slightly elevated, indicating the thyroid gland is producing normal amounts of thyroid hormone [1]. However, in toxic MNG patients, TSH levels are suppressed because the goiter is autonomously producing excess thyroid hormone, and the pituitary gland, due to negative feedback, reduces TSH to try to compensate for this overproduction [1]. 

A thyroid ultrasound is generally used for the initial evaluation of thyroid nodules and may also detect an increased risk of malignancy [1]. The Thyroid Imaging Reporting and Data System (TI-RADS) was designed to decrease the need for biopsies and to improve overall diagnostic accuracy [3]. Five ultrasound features of thyroid nodules (composition, echogenicity, shape, margin, and punctate echogenic foci) are given points and added to determine the level of malignancy, ranging from TR1 (benign) to TR5 (highly suspicious) [3]. At TR3 (mildly suspicious), an FNA is performed if the nodule is greater than 2.5 cm [3]. An FNA is considered the gold standard and the best diagnostic test for determining the malignancy of a thyroid nodule due to its high diagnostic sensitivity [4]. The standard classification for the FNA is the Bethesda system, which ranges from Category I (non-diagnostic) to Category VI (malignant) [5]. Each category has a specific risk of malignancy associated with it, aiding in guiding physicians on next steps [5]. 

After an ultrasound or FNA, patients are presented with appropriate options for further management. Often, a lobectomy is recommended for lower-risk thyroid cancer patients [6]. Higher-risk patients may require a total thyroidectomy to improve patient outcomes and reduce the possibility of recurrence [6]. High-risk patients may present with a large tumor size, tumor characteristics such as extrathyroidal extension (ETE) or multifocality, lymph node metastasis, etc. [6]. However, research shows that the initial choice of surgical treatment plays an important role in future patient outcomes [6]. In patients with risk factors such as older age (over the age of 45), larger tumors, and certain aggressive features of papillary/follicular thyroid carcinoma, metastasis is more common. A total thyroidectomy may not account for this, highlighting the importance of consistent follow-up appointments [7]. 

This case discusses a seemingly normal diagnosis of MNG leading to papillary thyroid carcinoma (PTC); however, complications arose after total thyroidectomy when ETE with possible lung metastasis was noted. Consistent follow-up appointments and proper detection tools were necessary to ensure proper monitoring of the post-operative patient. 

## Case presentation

A 71-year-old male presented for a comprehensive endocrine consultation, with incidental findings of MNG. The patient complained of voice weakness, difficulty swallowing, and dyspnea, with symptoms progressively worsening over time. He had no family history of thyroid disease, denied a history of radiation, and maintained a good general state of health. His social history showed that he was a former smoker but was unremarkable in regard to exercise, diet, stress levels, and medical/surgical history. 

The differential diagnosis for a patient presenting with voice weakness, difficulty swallowing, and dyspnea warrants a comprehensive evaluation for possible malignant etiologies, with further testing required to confidently ascertain malignancy.

**Figure 1:**
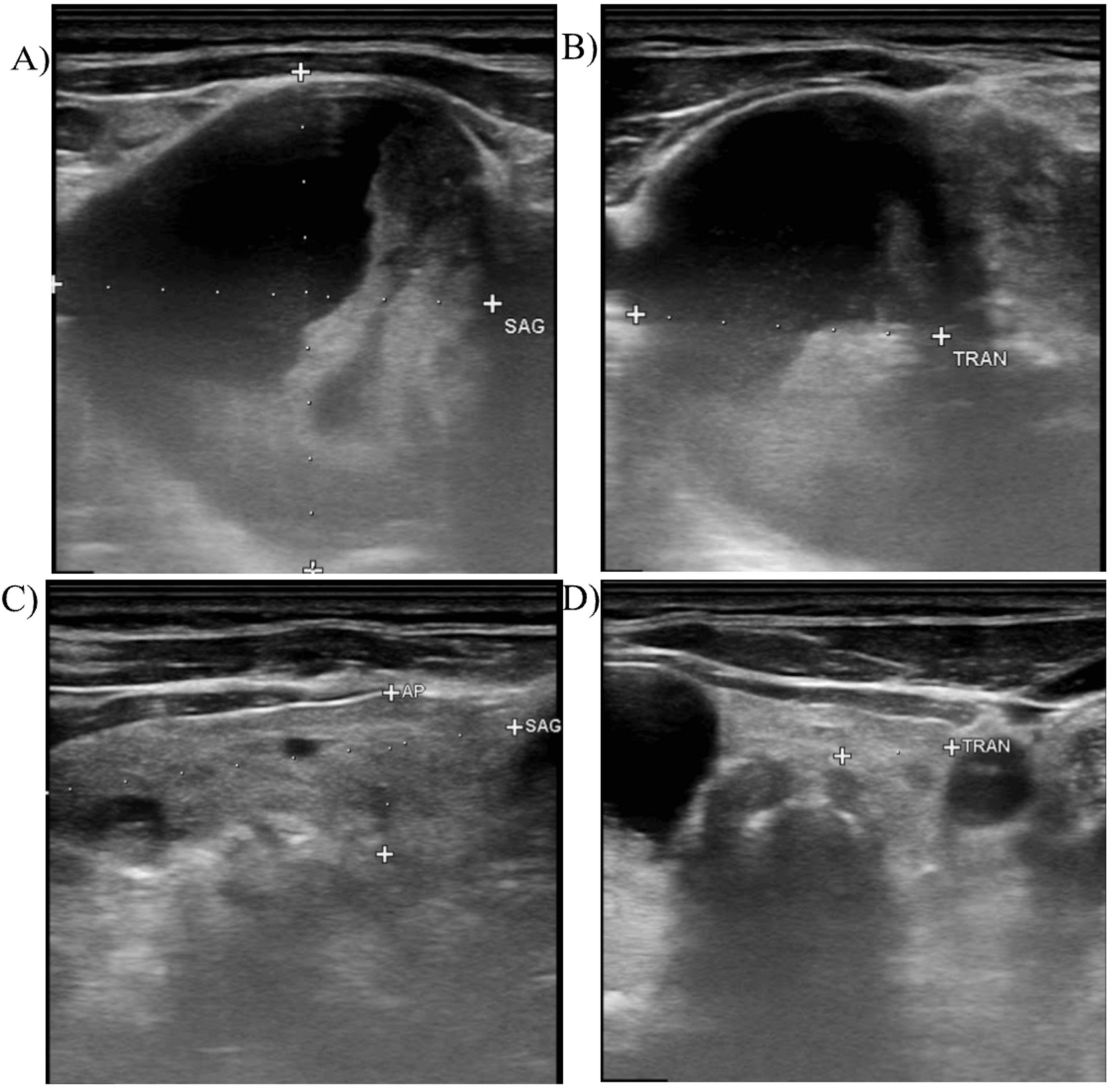
Sonograms of right and left lobes A and B depict the right lobe, C and D depict the left lobe. B and D depict the right and left lobes in a transverse view, respectively, and A and C depict the right and left lobes in a sagittal view, respectively.

**Figure 2:**
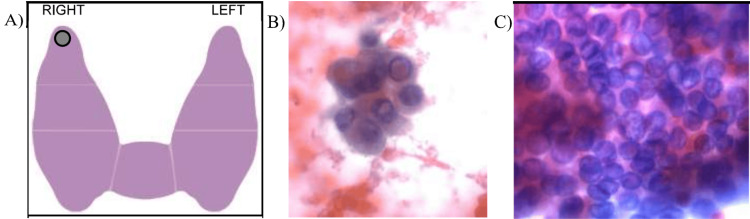
Fine-needle aspiration biopsy imagery A depicts the photomicrograph, specifying where the specimen was taken from; B depicts the right upper pole with intranuclear inclusions; and C depicts the same right upper pole with ovoid nuclei with grooves.

Thyroid function testing revealed a euthyroid state with TSH levels initially at 1.9 mIU/L. The thyroid peroxidase (TPO) and thyroglobulin (Tg) antibody status was unknown at presentation. A thyroid ultrasound (Figure 1) was performed with visualization of bilateral thyroid nodules, both over 1 cm in size, meeting the criteria for an FNA biopsy. The right and left poles proved to be heterogeneous, with the right lobe measuring 10.9 x 2.8 x 4.5 cm and the left lobe measuring 4.3 x 1.0 x 4.3 cm. Both lobes showed multiple cystic nodules, and the isthmus measured 0.3 cm. The thyroid proved to be asymmetric, with an enlarged right lobe and a deviated trachea by 2.5 cm to the left. Both lobes appear diffusely heterogeneous in echotexture, and echogenicity and vascularity were normal. This ultrasound was reported at a TI-RADS level 4 (moderately suspicious), and an FNA was performed on the largest right nodule (Figure 2). FNA results showed cause for concern, coming back as a Bethesda category 5 with the right upper pole suspicious for malignancy, and the endocrinologist diagnosed PTC with cystic changes. The patient was referred to a surgeon, where he underwent a total thyroidectomy. After the surgery, he was prescribed levothyroxine. 

The post-surgery report discussed the patient's anatomical pathology, claiming a PTC of a tall cell subtype at T4a staging, known to be an aggressive variant. Additionally, ETE to the trachea and lymphovascular invasion were reported, indicating a high risk for recurrence. On follow-up one month later, blood work was drawn, and it was discovered that the patient had a high TSH level of 47.20 mIU/L, a low free T4 level of 0.25 ng/dL, and detectable Tg antibodies (Table 1). Suspicion of metastasis increased due to the presence of Tg antibodies, and a whole-body scan (WBS) using I-131 without thyrogen was requested. The radiologist noted a suspicious left upper lung nodule and stated a differential including a primary lung tumor versus metastasis, as well as benign etiologies, recommending further evaluation to proceed. The patient was under close monitoring by the endocrinologist to ensure metastasis would be caught if it occurred. Follow-up appointments were scheduled every two months, and blood drawn prior to each appointment showed the patient’s TSH and free T4 stabilizing within six months of the surgery. A WBS taken one year after the initial one demonstrated no suspicion of metastasis; however, it is noteworthy to mention that the ETE visualized was unusual for a non-metastatic patient. 

**Table 1:** Free T4, Tg antibodies, and TSH blood lab values throughout treatment consolidated TSH: thyroid-stimulating hormone; WBS: whole-body scan

	Free T4 (ng/dL)	Thyroglobulin (Tg) Antibodies (ng/mL)	TSH (uIU/mL)
Normal Ranges	0.62 - 1.60	1.59 - 50.03	0.45 - 5.33
Initial Presentation	Unknown	Unknown	1.9
Post Surgery	0.25	0.05	47.20
Post WBS (3 months later)	0.94	0.05	1.64

The preoperative diagnosis was MNG with a PTC found upon FNA biopsy. After the surgery, metastasis was a concern, with the radiologist being unsure about whether the findings were a primary lung tumor or possibly of benign etiologies, leading to an indeterminate conclusion until further imaging was done. Herein lies the issue of differentiating between a true distant metastasis and incidental lung findings in a patient with high-risk thyroid cancer.

## Discussion

Multinodular goiter and cancer risk

The initial incidental findings of MNG led to the discovery of thyroid cancer and a concern for possible metastasis in the patient. Interestingly, there has been controversy in the literature about the risk of thyroid cancer in patients with toxic MNG and nontoxic MNG. Initially, studies have shown that MNG patients carried a lower risk of thyroid cancer; however, more recent studies have shown a higher risk of cancer (10-20%) in these patients [8]. A study by Cerci C. et al. with 294 patients operated on between 2001 and 2005 showed a thyroid cancer incidence rate of 10.58% in patients with nontoxic MNG and 9% with toxic MNG [9]. There was also a significant association with patients who are of younger age, male sex, and present nodular thyroids (p < 0.05) [8]. Several mechanisms have been proposed to explain this elevated risk. Long-standing MNG may expose thyroid tissue to chronic TSH stimulation, oxidative stress, and cycles of hyperplasia and involution, all of which can promote genetic instability within nodules. Nodules within an MNG also vary in autonomy and function, creating microenvironments conducive to clonal selection and neoplastic transformation.

In this case, the patient’s nontoxic MNG and subsequent discovery of carcinoma are consistent with these evolving epidemiologic patterns. The background of multinodularity likely contributed to diagnostic complexity and underscores the importance of vigilant evaluation of nodular goiters even in the absence of overt hyperthyroidism.

Clinical significance of extrathyroidal extension

Concern for metastasis increases once ETE is noted. While metastasis of thyroid cancer in lymph nodes is fairly common, ETE is less common, only occurring in about 23.5% of all PTCs [10,11]. ETE is defined as possible tumor extension beyond the thyroid capsule into the adjacent soft tissue [10]. Further divided into two subcategories, minimal/microscopic ETE or extensive/gross ETE, it is typically established through imaging or during an operation (such as total thyroidectomy) [10]. While with minimal ETE the percent recurrence is approximately 3 - 9%, gross ETE reports a percent recurrence of 23 - 40% [10]. 

In the present case, ETE was suspected on preoperative imaging but not ultimately identified in the final pathology report. This discrepancy highlights a known challenge in thyroid cancer staging; radiologic features suggesting capsular disruption or perithyroidal invasion may reflect inflammatory or fibrotic changes rather than true invasion. The absence of confirmed ETE in this patient lowers the risk classification and favorably impacts long-term prognosis.

Importantly, this patient was diagnosed with the tall cell variant of PTC, a subtype characterized by increased aggressiveness, higher rates of ETE, and an elevated likelihood of distant metastasis compared to classic PTC, which typically affects older patients. Awareness of this variant is critical, as it generally warrants more intensive postoperative surveillance.

Post-thyroidectomy management also includes TSH suppression, particularly in intermediate- to high-risk patients [1]. According to current guidelines, TSH should be suppressed to <0.1 mIU/L for high-risk disease and maintained slightly above this threshold for intermediate-risk categories [1]. Given the presence of a tall cell variant and suspicion of ETE, appropriate TSH suppression is essential in reducing recurrence risk in this patient.

Metastasis, diagnostic uncertainty, and prognostic implications

In the case presented above, the patient was diagnosed with PTC, with the most common form of treatment being total thyroidectomy. The surgery was successful; nevertheless, abnormal lab results led to further concern with a WBS, leading to suspicion of metastasis into the lung. A similar case report described a female found to have nodules in both lungs upon examination, and further imaging using computer tomography (CT) scans led to indeterminate findings, as seen in the aforementioned case [12]. After undergoing thoracoscopic left lower lobe wedge resection, the postoperative diagnosis confirmed a metastatic carcinoma in the left lower lung mass, and a final diagnosis of lung metastasis of thyroid cancer was concluded [12]. Although thyroid cancer is the most common form of head and neck cancer, distant metastasis from it is rare and is only diagnosed in 1 - 4% of patients [12]. If suspicion of metastasis is noted, close monitoring of the patient is necessary since these types of cases usually have a poor prognosis and are the leading cause of thyroid cancer-related deaths.

Current guidelines recommend a stepwise approach when metastasis is suspected but not confirmed, including serial imaging, consideration of high-resolution CT, PET/CT in selected cases, and tissue biopsy if the lesion is accessible and demonstrates growth or concerning features. Repeat radioactive iodine (RAI) therapy may be considered in cases with rising thyroglobulin levels, iodine-avid lesions, or progression on surveillance imaging.

In this patient, the lack of definitive evidence of metastasis emphasizes the importance of close monitoring, serial imaging, and biochemical surveillance. The coexistence of the tall cell variant, an aggressive histologic subtype, with suspected but unconfirmed pulmonary involvement requires careful long-term follow-up to ensure early detection of recurrence or progression.

## Conclusions

This case underscores the importance of recognizing aggressive variants of PTC, particularly the tall cell subtype, which carries higher risks of extrathyroidal invasion, recurrence, and distant spread. Although ETE was suspected on imaging in this patient, it was not confirmed on final pathology, and the suspected pulmonary metastasis remained indeterminate. This highlights the diagnostic challenges clinicians face when radiologic findings suggest metastatic disease that cannot be definitively established.

By originating in the context of MNG and demonstrating features of an aggressive histologic variant, this case contributes to the growing literature on the diverse presentations and progression patterns of thyroid cancer. It emphasizes the need for individualized, risk-adapted surveillance strategies that incorporate tumor subtype, patient characteristics, and biochemical trends. Long-term follow-up, including periodic imaging, thyroglobulin monitoring, and appropriate TSH suppression, is essential for early detection of recurrence or metastatic progression. Multidisciplinary management, including ongoing coordination between endocrinology, surgery, radiology, and nuclear medicine, is critical for optimizing outcomes and determining the role of further interventions such as RAI therapy.

Ultimately, this case highlights the complexity of diagnosing and managing aggressive variants of PTC and reinforces the importance of structured, long-term surveillance in patients at elevated risk for recurrence or metastasis.
